# From CPAP to tailored therapy for obstructive sleep Apnoea

**DOI:** 10.1186/s40248-018-0157-0

**Published:** 2018-12-03

**Authors:** Kate Sutherland, Kristina Kairaitis, Brendon J. Yee, Peter A. Cistulli

**Affiliations:** 1 Charles Perkins Centre, The University of Sydney, Sydney, Australia; 2 Faculty of Medicine & Health, The University of Sydney School of Medicine, Sydney, Australia; 3 Centre for Sleep Health & Research, Department of Respiratory Medicine, Royal North Shore Hospital, Northern Sydney Local Health District, Sydney, Australia; 4 Ludwig Engel Centre for Respiratory Research, Westmead Institute for Medical Research, University of Sydney, Sydney, Australia; 5 Department of Respiratory and Sleep Medicine, Westmead Hospital, Sydney, Australia; 6 NHMRC Centre for Integrated Research and Understanding of Sleep (CIRUS) and NHMRC NeuroSleep Centre Woolcock Institute of Medical Research, Sydney, Australia; 7 Department of Respiratory and Sleep Medicine, Royal Prince Alfred Hospital, Sydney, Australia

**Keywords:** Obstructive sleep Apnoea, Treatment, Continuous positive airway pressure, Personalised medicine, Phenotyping

## Abstract

Obstructive Sleep Apnoea (OSA) is a common sleep disorder that is associated with daytime symptoms and a range of comorbidity and mortality. Continuous Positive Airway Pressure (CPAP) therapy is highly efficacious at preventing OSA when in use and has long been the standard treatment for newly diagnosed patients. However, CPAP therapy has well recognised limitations in real world effectiveness due to issues with patient acceptance and suboptimal usage. There is a clear need to enhance OSA treatment strategies and options. Although there are a range of alternative treatments (e.g. weight loss, oral appliances, positional devices, surgery, and emerging therapies such as sedatives and oxygen), generally there are individual differences in efficacy and often OSA will not be completely eliminated. There is increasing recognition that OSA is a heterogeneous disorder in terms of risk factors, clinical presentation, pathophysiology and comorbidity. Better characterisation of OSA heterogeneity will enable tailored approaches to therapy to ensure treatment effectiveness. Tools to elucidate individual anatomical and pathophysiological phenotypes in clinical practice are receiving attention. Additionally, recognising patient preferences, treatment enhancement strategies and broader assessment of treatment effectiveness are part of tailoring therapy at the individual level. This review provides a narrative of current treatment approaches and limitations and the future potential for individual tailoring to enhance treatment effectiveness.

## Introduction

**Fig. 1:**
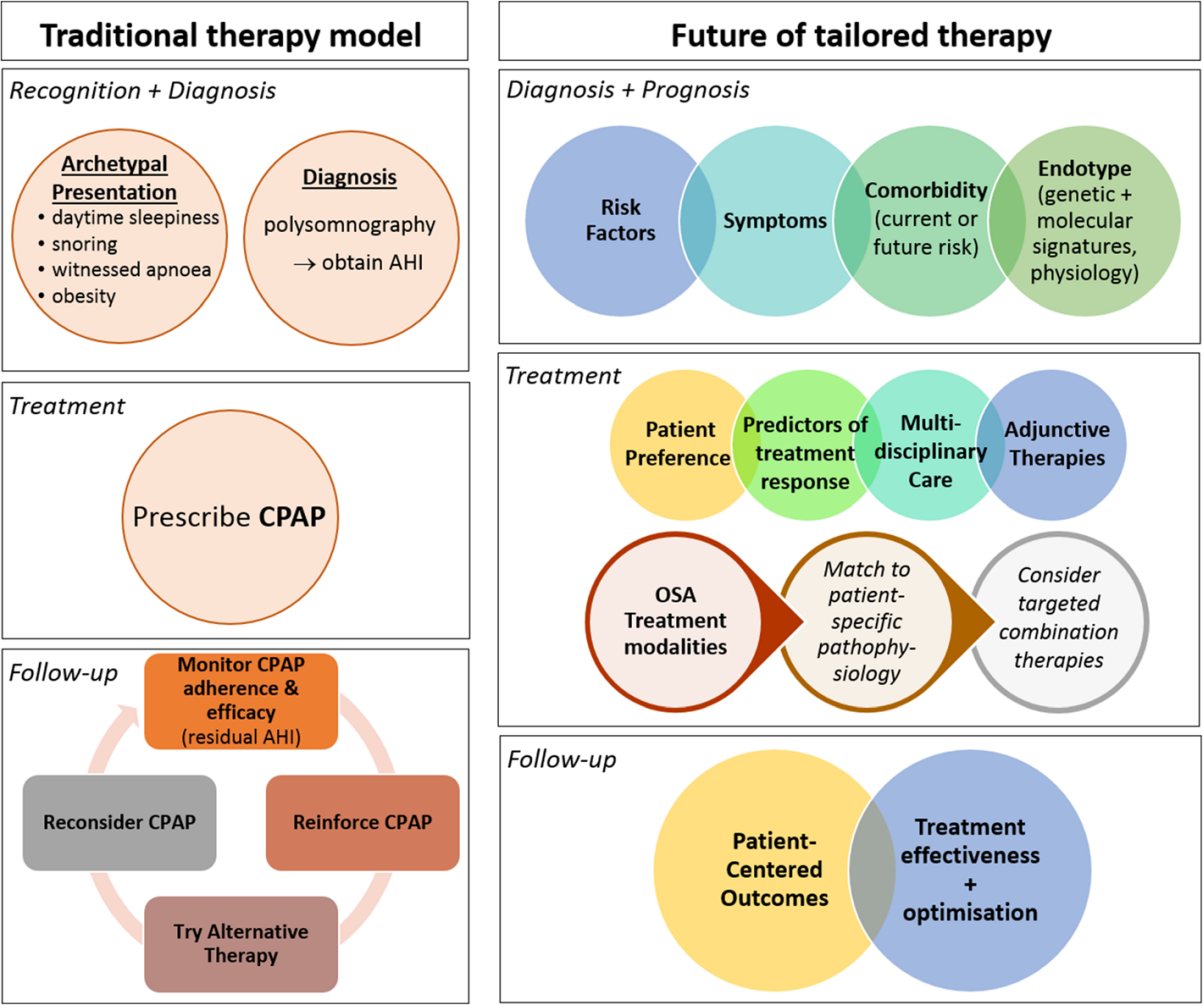
The future of tailored therapy for Obstructive Sleep Apnoea (OSA). The left panel shows the traditional clinical pathway for recognition and treatment of OSA The right panel shows a proposed new approach for the future of tailored therapy. A range of clinical expression subtypes are recognised (including asymptomatic). Diagnosis provides more information than a single metric such as the AHI. Prognostic information on susceptibility to future comorbidity risk from biomarkers. Information about endotype from more sophisticated analysis of genomic and molecular signatures, as well as physiological signals (also help to guide OSA treatment selection). OSA treatment informed by general factors of patient preference, predictors of treatment response, multidisciplinary care for adjunctive therapies (to address symptoms and co-morbidities). Follow up focus on patient-centred outcomes (not just efficacy assessment) + optimisation

Obstructive Sleep Apnoea (OSA) is a common sleep disorder, characterised by repetitive obstruction of the pharyngeal airway during sleep which leads to intra-thoracic pressure swings, sleep fragmentation, and intermittent hypoxia. These cyclical perturbations provide a mechanistic framework for the association of OSA with a range of clinical morbidity including metabolic and cardiovascular disease, cognitive impairment, cancer and increased rates of mortality [1–4]. Prevalence studies suggest some level of sleep-disordered breathing can be detected at rates of 9–38% in general populations [5]. Furthermore, ongoing epidemiological studies suggest prevalence rates have increased over the last 20 years in the order of 14–55% depending on severity, gender and age group [6]. Increasing prevalence could be largely attributable to increasing obesity rates, as obesity is a well-known risk factor for OSA. Other common risk factors for OSA include craniofacial abnormalities, male gender, and family history. However, OSA is increasingly appreciated as a heterogeneous disorder with individual differences in risk factors, clinical expression and potential consequences of the disease. There is value in characterising individual subtypes within a disease to identify targeted therapeutic approaches to enhance patient outcomes, as has been demonstrated in other heterogeneous disorders, such as asthma, COPD and cancer [7–9]. Personalised medicine is an emerging goal in many fields and has been distilled into the concept of P4 medicine; the four P’s being Prediction, Prevention, Personalisation, and Participation [10]. This framework ultimately aims to advance healthcare such that disease can be predicted before it manifests, thereby creating the potential for prevention. Additionally, individuals are informed and involved in their own health and participate in their own health care decisions and monitoring of outcomes. Finally, in the event of disease, treatments are personalised to an individual in order to maximise outcomes. There are many opportunities to tailor therapy for OSA, including understanding of different presentations and susceptibility to future risk, tailoring therapy to pathophysiology and patient preference, and follow-up focused on health outcomes. These possibilities are contrasted with the 'traditional' therapy model in Fig. 1, with these future opportunities discussed in this review.

Personalisation of treatment is particularly important in OSA. As a chronic condition, effective therapy is required over the long-term to promote good health and wellbeing. This requires a therapy that is accepted by the patient, effectively controls the disorder, and is adequately adhered to. The standard of therapy has long been Continuous Positive Airway Pressure (CPAP) devices. CPAP can be viewed as a ‘one size fits all’ solution to OSA, as when applied CPAP prevents OSA regardless of the underlying mechanisms of apnoea and site of pharyngeal collapse, or patient phenotype. There are different pathways to OSA with both structural and physiological risk factors that differ in relative importance between individuals. Structural risk factors include craniofacial structure, enlarged upper airway soft tissues and obesity. Physiological risk factors such as ventilatory control system abnormalities or ineffectiveness of dilator muscle output can also contribute to OSA. Individuals with OSA have their own unique combination of pathophysiological mechanisms that contribute to disease. Knowledge of these provides a means to tailor therapy to an individual.

## Continuous positive airway pressure

Since its first report in 1981 [11], CPAP therapy for OSA rapidly became the first-choice therapy of symptomatic OSA. To this day, it continues to be the treatment of first choice by most sleep medicine practitioners, largely because it is universally successful in preventing upper airway obstruction. CPAP works by counteracting collapse and narrowing by pneumatically splinting the upper airway via the application of positive pressure. The acute efficacy of CPAP therapy is highly evident during CPAP titration sleep studies, during which the pressure is incrementally increased until all evidence of obstruction and resultant intermittent hypoxic episodes are abolished. CPAP therapy is highly efficacious in returning the apnoea-hypopnea index (AHI) back to the normal range, usually taken as < 5 events/hour.

The diagnosis of clinically significant OSA is established by the combination of clinical assessment and a diagnostic sleep study, the latter most often an in-laboratory polysomnogram (PSG) or a home sleep study. This should be followed by a discussion between practitioner and patient (and often patients’ partner) that focuses on the individual’s risk factor(s) for the occurrence of OSA and to the presence of significant co-morbidities that may be exacerbated by OSA. Such potentially modifiable risk factors include obesity, nasal and other upper airway pathology, and aggravating lifestyle issues such as smoking and excessive alcohol use. These factors need to be addressed in parallel with direct therapy, used to abolish the pathophysiological events of OSA.

Good evidence has accumulated that effective CPAP improves the neurobehavioral and cardiovascular consequences of OSA [12, 13]. Studies confirm that sleepiness and some of its consequences, such as the incidence of sleepiness-related motor vehicle accidents, are improved with CPAP. Residual sleepiness on CPAP is recognised, and adjunctive therapy with stimulant medication has been proposed [14]. In many studies, general measures of quality of life in sleepy OSA patients are also improved by use of CPAP. Adverse cardiovascular consequences of untreated significant OSA, in particular systemic hypertension but also some other measures of cardiovascular morbidity, and mortality, appear to be improved by CPAP. However, even with good adherence, not all patients will experience health benefits from CPAP therapy. This has been demonstrated for blood pressure, for which there are patients with good adherence who do not achieve blood pressure reduction [15]. Studies suggest that factors such as the pattern of nocturnal blood pressure dipping and heart rate might be clinical predictors, although these factors only explain about one third of the variability in the blood pressure response to CPAP treatment [16]. Recently, molecular signatures have been explored as potential predictors of blood pressure response to CPAP treatment in OSA patients with resistant hypertension, and a cluster of three micro-RNAs discriminated responders and non-responders [17]. Hence, there is potential for blood biomarker profiles to be developed for predicting different aspects of treatment response.

Importantly, not all patients with OSA accept or are able to tolerate CPAP. Even amongst sleepy patients with moderate -severe OSA adherence to prescribed therapy (defined as > 4 h average nightly use) is poor with only 46–83% being adherent [18]. A number of individual factors may indicate the likelihood of inadequate adherence to CPAP therapy, including increased nasal resistance, claustrophobia, and other psychological factors, as well as machine related factors (eg. mask leak). This limits the ‘real world’ effectiveness of CPAP, and highlights the need for alternative therapies.

## Non-CPAP therapy alternatives

There is an ongoing need to consider alternative therapeutic options. Most current alternative therapy strategies have a common theme in that they are not completely efficacious in all OSA patients. Therefore, a comprehensive understanding of the individual patient phenotype is needed in order to select the most appropriate therapy and to maximise clinical benefit.

### Weight loss

Obesity is the strongest reversible risk factor for developing OSA. Large epidemiological studies have shown a strong relationship between weight gain and developing OSA [19–21]. Up to 40–60% of obese subjects suffer from OSA [22]. OSA susceptibility is determined by pharyngeal collapsibility as a consequence of elevated critical closing pressure. Both mechanical (adipose deposition in peripharyngeal fat pads) and neural factors (depressed neuromuscular control of upper airway) determine critical closing pressure [23, 24]. Given that a 10% weight gain was observed to be associated with a 32% increase in OSA severity and a 10% weight loss was associated with a 26% reduction in AHI, the importance of weight loss and lifestyle modification should not be underestimated [20]. Randomized controlled studies have shown weight loss in obesity is effective in lowering OSA severity and reducing cardiovascular risk [25–27]. Weight loss with very low energy diets (VLED) is an effective way in reducing weight, both rapidly and significantly, in OSA [25]. However, maintenance of weight loss after 6 months is challenging [28]. Recently, a study using maintenance diets following a VLED was successful in prolonged weight loss and improvements in OSA up to a year [29]. The effectiveness of weight loss therapy depends on initial OSA severity. Even in mild to moderate OSA, medical weight loss (up to 10%) leads to both symptomatic and metabolic improvements. However, cure of OSA (AHI < 5 events/hour) occurs only in a minority of subjects [30]. In severe OSA, the benefits of weight loss (either medical or surgical) on OSA are less certain. Although some will experience substantial reductions in AHI, the majority will still have ongoing moderate to severe OSA and may still require adjunct CPAP [30, 31].

Despite this, weight loss leads to significant sleep related symptomatic improvements that may be independent of AHI changes [31]. Weight loss also improves quality of life, cardiovascular and metabolic health independent of changes in AHI. It should also be emphasized that improvements in OSA severity with lifestyle interventions can be sustained over 1–4 year periods despite weight regain [29, 32]. The combination of both weight loss and CPAP, although not extensively studied, has been shown to have potential synergistic benefits on cardiometabolic factors [33]. In terms of predictors of success of weight loss therapy for OSA treatment there is limited information on which individuals should be targeted for weight loss. Craniofacial skeletal structure may be an indicator of effectiveness of weight loss. Two studies have identified smaller, or more restricted, maxillary and mandibular measurements as an indicator of a better response to weight loss in terms of AHI reduction [34, 35]. A smaller craniofacial boundary likely increases the impact of regional adiposity in increasing critical closing pressure [36].

### Positional therapy

Positional therapy aims to prevent sleep in the supine position in which sleep-disordered breathing is often more severe. There are a number of devices designed for this purpose, ranging from a tennis ball affixed to the back of a pyjama top to new generation electronic devices, which utilise increasing vibrations from sensors placed on the back of the neck or chest to prompt a move away from the supine body position. Studies of positional therapy from tennis balls to commercial devices have generally shown improvement in AHI but studies are limited to small RCTs and case series [37]. Minimal difference in reduction in supine sleep time and improved PSG indices between a commercial waistband positioner or self-made tennis ball type solutions have been observed short-term (~ 12 weeks) [38]. However, sleep quality metrics improved more using a new generation positional sensor device compared to the tennis ball technique [39]. A recent meta-analysis has looked specifically at new generation positional devices [40]. On average, greater than 50% reduction in total AHI and over 80% reduction in supine sleep time were reported across 6 studies of these devices. These new generation positional devices have objective monitoring capabilities and adequate compliance (defined as ≥4 h/night on ≥5 days/week) in 75.9% of patients over the first month of treatment [39]. However, long-term adherence to positional therapy is not well understood, and little is known about long term impact on health outcomes. Subjective reports indicate that after 1 year, 65% of patients report they are no longer using position therapy despite good initial compliance to therapy [38].

The phenotype of OSA patient suited to positional therapy is clearly one for whom apnoeas and hypopneas occur primarily in the supine sleep position, whereas other positions are less affected. Position OSA (P-OSA) is reported in the range of 50–60% of patients undergoing PSG in sleep clinics, with supine-isolated OSA present in 25–30% [41]. Therefore, there is a significant proportion of OSA patients for whom positional therapy could be the primary therapy. Positional therapy may additionally be a beneficial adjunct to upper airway surgery [42] or oral appliance therapy [43] to reduce AHI across total sleep time.

### Oral appliances

Oral appliance therapy aims to reposition craniofacial or intraoral structures in order to increase the pharyngeal airway space and prevent pharyngeal collapse. Oral appliances include tongue retaining devices which aim to hold the tongue in a more anterior position [44]. However, the largest evidence base and guidelines exist for mandibular advancement appliances (OA_m_) [45]. OA_m_ are dental devices, which attach to the upper and lower dental arches in a configuration to protrude the lower jaw relative to the upper jaw. OA_m_ exist in numerous designs which vary in the amount of customisation to the dentition, fabrication material, amount of occlusal coverage, whether the device consists of a single plate (monobloc) or two separate plates, the amount of vertical mouth opening permitted, the advancement mechanism and ability to adjust advancement level (titration). There are limited studies directly comparing different OA_m_ designs but guidelines recommend a customised and titratable device as best practice [45]. OA_m_ significantly reduce sleep disordered breathing metrics and snoring compared to placebo oral devices (which provide no mandibular advancement) [46–48]. OA_m_ reduce AHI by an average of around 50% [49], and 30–70% of patients achieve a complete response (treatment AHI < 5 events/hour) [50]. Conversely, there are around a third of patients who will have less than 50% reduction in AHI, in whom clinical benefit is questionable.

There are a number of patient characteristics which have been associated with a favourable outcome of OA_m_ therapy. These include less severe OSA (lower AHI), less obesity (smaller neck circumference, lower BMI) and younger age. However, these characteristics represent a guide only, and there are no accurate thresholds for any of these factors to exclude patients from therapy [49]. Craniofacial structure can also play a role in effectiveness of OA_m_ therapy. Most studies investigating craniofacial structures associated with treatment responsiveness have used lateral cephalometric x-rays, a two-dimensional analysis of the facial profile. There are commonly reported characteristics associated with treatment response including mandibular plane angle, hyoid position, size of upper airway soft tissue and cranial base angle [51]. However, as recently summarised in systematic review, there is large variation in the relatively small studies of craniofacial structure, including differences in measurements taken and treatment response definitions [51]. Therefore, there is not a confirmed set of measurements indicating good treatment outcome, and craniofacial characteristics alone are not likely to be a robust patient selection tool [52].

Many prediction methods have been proposed to select patients for OA_m_ therapy, which have varying degrees of clinical applicability, predictive accuracy and validation [53]. For a recent review see [53]. Most studies using indirect measures to predict pharyngeal response to mandibular advancement have not withstood validation. The front-runner is currently a direct assessment during sleep using remote-controlled mandibular protrusion [54, 55]. Other methods to assess the effects of mandibular advancement on the pharyngeal airway include Nasopharyngoscopy applied either during wakefulness [53] or drug-induced sleep (DISE) [56] and show promise but require validation in other samples. Inability to accurately predict which patients will not receive therapeutic benefit from OA_m_ is a clinical barrier.

### Upper airway surgery

Upper airway surgery aims to improve anatomy to prevent pharyngeal collapse. Upper airway anatomical impairment can be modelled as an imbalance between the soft tissues enclosed within the maxillo-mandibular bony enclosure [36]. Upper airway surgery can reduce *soft tissues* (eg, uvulo-palatopharyngoplasty, tongue reduction, adeno-tonsillectomy) or increase the size of the bony enclosure (eg *maxillomandibular surgery*), repositioning of the hyoid bone (hyoid repositioning), or increasing nasal patency (eg turbinate reduction surgery) [57].

#### Soft tissue surgeries

In children, adeno-tonsillectomy is accepted as first line therapy for OSA. In adults, surgery for OSA previously involved a single procedure such as uvulopalatopharyngolasty (UPPP), which reduces upper airway collapsibility [58]. Modern surgical approaches are usually offered to subjects who cannot tolerate CPAP, or to improve CPAP usage by improving nasal patency [57]. Current approaches to surgery usually take a multi-level approach, with incremental surgical procedures such as a UPPP combined with tongue reduction [57]. A Cochrane review in 2005 concluded that surgery could not be recommended as first line treatment for OSA [59], although some subgroups of patients benefit from surgery. A more recent single centre randomised control trial demonstrated that modified UPPP may be effective in selecting patients [60], with improvements in blood pressure [61], as well as reductions in sleepiness and improvements in quality of life [60].

#### Maxillomandibular surgery

Meta-analysis of trials of maxillo-mandibular surgery also concluded that there were improvements in sleepiness symptoms, and OSA severity in subjects who had failed other therapies including upper airway surgery [62]. Maxillo-mandibular surgery has been demonstrated to be effective, with an initial improvement in 80% of subjects [63]. Maxillo-mandibular surgery is more effective in younger subjects, with lower BMI, and those with a higher AHI [62, 63]. Cephalometric characteristics may also be predictive of success [64]. Furthermore, a retrospective review concluded that these benefits were maintained for more than 12 years [64].

Selecting the right patient for various upper airway surgical procedures is complex. An initial step may be to determine that their OSA relates largely to anatomical pathophysiology [65]. Knowledge of the site and nature of pharyngeal collapse could also help to predict outcome. Drug Induced Sleep Endoscopy (DISE) has been used as a method to identify predictors of surgery for OSA. More complete or severe concentric collapse at the palatal level or anteroposterior collapse at the tongue base or epiglottis observed while under sedation has been associated with non-response to subsequent surgery (any combination of palatal, tongue radiofrequency ablation and hyoid suspension) [66].

### Hypoglossal nerve stimulation

Surgery can also target effectiveness of upper airway dilator muscles through Hypoglossal nerve stimulation (HGNS). HGNS is a relatively new approach to treat upper airway collapse that surgically implants a cuff around the hypoglossal nerve attached to an electrical pulse generator in the chest. These devices stimulate the hypoglossal nerve resulting in protrusion of the tongue during respiration. Current devices have a sensor for respiratory efforts and stimulate the hypoglossal nerve to increase tongue protrusion in time with inspiration [67, 68]. In patients with a low BMI, who are intolerant of CPAP, these devices have demonstrated success with reductions in AHI, improvements in oxygen and improvements in sleepiness in cohort studies [68]. These improvements are maintained after 3 years [69]. Withdrawal of hypoglossal nerve stimulation results in a return of symptoms and obstructive events, supporting hypoglossal nerve stimulator as the mechanism for improvement [70]. Hypoglossal nerve stimulation has not yet been demonstrated to result in long term improvements in vascular outcomes, and again remains limited to a therapeutic option in some CPAP intolerant subjects. Complete and concentric collapse in the palatal region during DISE has been identified as a negative predictor for response to hypoglossal nerve stimulation [71].

### Emerging therapies

Studies designed to delineate the various contributors to recurrent upper airway closure during sleep identify anatomy, muscle responsiveness, arousal threshold, or respiratory system control as important [72, 73]. A careful analysis of the contributors to OSA in 75 subjects found 80% had anatomical impairment [74], however this was modified by other factors, including upper airway muscle responsiveness, the arousal response, and respiratory control [74].

A low arousal threshold, or propensity to wake easily in response to a disturbance is thought to be important in perpetuating repetitive obstructive events in subjects with OSA by promoting instability around the sleep/wake state [72, 73]. To counter this, some investigators have trialled sedatives as a therapy for OSA, aiming at reducing arousal threshold. Physiological investigations have demonstrated that sedative agents can increase arousal threshold [75–77], however no trial has convincingly demonstrated an improvement in severity of sleep disordered breathing with sedatives [76, 78]. Currently, sedative therapy to treat obstructive sleep apnoea does not have evidence to support a benefit, even in subjects selected to have a low arousal threshold [76].

Pharmaceutical approaches to increasing upper airway activity have also been trialled. Desipramine, an agent which stimulates the noradrenergic neurons can increase muscle response, and reduce upper airway collapsibility [79, 80], although this had no overall effect on OSA severity [80]. Further investigations of drugs that target upper airway muscle response are in progress.

Approaches aimed at manipulating respiratory control have also been trialled as treatment for OSA. The aim of this therapy is to alter the “Loop Gain” of the respiratory control system. Loop gain quantifies respiratory system response to a perturbation, with a loop gain greater than 1 indicating the response is greater than the disturbance, leading to a perpetuation of the disturbance. A meta-analysis of oxygen therapy in OSA concluded that oxygen therapy improves oxygen saturation and reduces AHI in subjects with OSA, but may also result in the prolongation of apnoeic events [81]. Measures of chemosensitivity to carbon-dioxide have been demonstrated to be important predictors of an individual response to oxygen therapy [82], and it may be that daytime measurements of ventilatory response could be used to personalise oxygen prescription for OSA.

Understanding of the contribution of anatomical impairment and individual non-anatomical contributors to OSA pathophysiology upper airway muscle responsiveness, arousal threshold, or respiratory control in individual patients could suggest success with these emerging treatment modalities, either singularly or in combination.

## Personalisation strategies and tools

### Optimising CPAP adherence

Considerable effort has gone into the development of strategies to enhance CPAP adherence, a critical goal for the attainment of health benefits. These include patient and devices factors. Early follow up by phone and direct clinic attendance enable confirmation of effective treatment use and trouble-shooting of any ongoing or evolving difficulties, and has been demonstrated to augment compliance. Cognitive behaviour therapy has been demonstrated to enhance self-efficacy and the adoption and adherence to CPAP. There is considerable scope to develop personalised approaches to the implementation of CPAP based on patient anatomical and psychological characteristics. For example, personalisation of mask fitting and addressing psychological barriers to treatment or to comorbid insomnia are feasible strategies depending on the patient phenotype.

The advent of automatic CPAP improved the efficiency with which CPAP could be implemented, with greater convenience for the patient by enabling implementation in the home setting. Such devices deliver appropriate but varying positive pressure breath-to-breath, and today most CPAP devices have electronic data storage cards that collect important adherence and efficacy data. The superiority of automatic CPAP over fixed-pressure CPAP is not yet proven, although they tend to provide greater convenience. More recently, cloud based technologies have led to the development of patient engagement tools that appear to be associated with better adherence [83]. There are ongoing advances in mask interface customisation, creating greater scope for personalisation.

There are also emerging “big data” opportunities in OSA, particularly through the advent of large-scale cloud based collection of CPAP adherence data. It is envisaged that this will make it possible to define adherence phenotypes, and ultimately using data analytics to identify predictors of treatment adherence. In this way we will be able to tailor interventions to optimise CPAP adherence.

### Phenotypic approaches to OSA

We are approaching an era where understanding patient phenotype will allow personalised strategies towards treatment. There is a recent move towards identifying novel phenotypes of patients using discovery or unsupervised machine learning analyses. These methods aim to describe hidden structure within data. An example of unsupervised machine learning is cluster analysis or clustering. Cluster analysis aims to group cases such that cases within the same group (or cluster) are most similar to each other than those in other groups. Clustering methodologies have recently been used to identify novel subtypes of OSA patients based on clinical characteristics. There is emerging evidence that phenotypes identified in this manner may have clinical meaning and ultimately may help to tailor therapy.

#### Clinical phenotypes

Clustering methods were applied to data from a self-reported symptom questionnaire in the Icelandic Sleep Apnea Cohort (ISAC, *N* = 822, moderate-severe OSA patients) [84] to elucidate clinical symptom subtypes with subsequent validation in an international cohort [85]. Results of the cluster analysis revealed 3 groups which could be described in terms of symptom presentation as either asymptomatic, excessively sleepy or disturbed sleep (insomnia symptoms). These three symptom groups did not differ in terms of AHI so cannot be explained by differences in disease severity. This suggests there are distinct patterns of OSA patients in terms of clinical expression. These 3 symptom clusters were again identifiable in a general population sample from South Korea [86], although in the asymptomatic subgroup made up over half of the sample. Collectively these studies suggest these symptom subtypes are widely applicable and are evident irrespective of ethnicity and study population and are not just an artefact of clinical referral patterns.

These clinical symptom subtypes have now been explored in relation to treatment outcomes in the ISAC cohort [87]. Patients were re-assessed after 2 years of CPAP treatment and comparisons made between the symptom subgroups identified at initial clinical presentation. In the disturbed sleep group changes in insomnia-related symptoms were similar between users and non-users of CPAP treatment. This suggests these individuals require additional targeted therapy to address the insomnia complaints in addition to CPAP. Therefore, knowledge of symptom phenotype has implications for tailoring treatment strategies.

Data driven discovery approaches have also been applied to clinical sleep study data [88] and associations with future cardiovascular disease with novel PSG clusters characterised by periodic limb movements, but not traditional AHI severity categories (mild, moderate, severe). Being able to identify patient subtypes at risk of future comorbidity has implications for tailoring therapy. If it was known that an OSA patient is particularly susceptible to risk, then additional effort is required to monitor adherence and ensure treatments with adequate long-term acceptability are available to the patient.

Other discovery approaches include universal assessment of what is going on at the level of the genes (genomics), mRNA (transcriptomics), proteins (proteomics) and metabolites (metabolomics). ‘OMICS’ approaches to characterise molecular signatures associated with OSA are of interest to develop diagnostic markers of disease but could also be used to ascertain disease risk and treatment responses [89].

#### Anatomical phenotyping

Anatomical phenotyping of craniofacial skeletal and soft tissue structures related to OSA have been performed by both two-dimensional (cephalometric x-rays) and three-dimensional (e.g. magnetic resonance imaging, cone beam computed tomography) imaging techniques [90]. Anatomical phenotyping using detailed image analysis has been used to understand response to various treatments such as oral appliance therapy [91], weight loss [92], upper airway surgery [93] and hypoglossal nerve stimulation [94]. Three-dimensional imaging, such as magnetic resonance imaging (MRI) or computed tomography (CT), have additionally been used to produce patient-specific anatomical models of the airway to provide the basis for computer simulations of airflow and pharyngeal collapse. These simulations based on accurate and patient-specific anatomy provides a non-invasive method of predicting how the airway is likely to behave with different forms of treatment. Computation fluid dynamics (CFD) is one method of modelling that has been applied to patient airway models with the view to being able to predict the likely outcome of oral appliance therapy [95, 96], soft tissue surgery (adenotonsillectomy) [97], and skeletal surgeries such as maxillomandibular advancement surgery [98].

These detailed imaging and simulation processes are complex and are currently beyond the scope of clinical practice for matching individual patients to particular therapies. Another approach is anatomical phenotyping using high-throughput and less expensive methods. For example, it has been proposed that a simple anatomical assessment in the Mallampati Score may be a reflection of anatomical balance [99] and this has been explored as a simple anatomical phenotype of OA_m_ response. Mallampati score may also be an indicator of the site of pharyngeal collapse, particularly retrolingual collapse [100]. A simplified method of quantitative assessment of facial phenotype from photographs has been developed in OSA patients which reflects OSA risk [101–103]. Surface facial dimensions appear to capture phenotypic information about underlying structures associated with OSA risk [104, 105] therefore this method may be a useful surrogate in genetic or epidemiological studies requiring large amounts of data. The method may be useful for identifying those with an anatomical phenotype of OSA pathogenesis and therefore may have use in treatment selection [106]. A quantitative photographic method for intraoral structures has also been developed [107].

#### Polysomnographic phenotyping

OSA is diagnosed by PSG. In the laboratory a full complement of signals includes airflow, oximetry, respiratory effort, ECG, EEG, EOG, EMG, and body position. In clinical practice this information is for the most part distilled into a single metric, the Apnoea-Hypopnoea Index, on which treatment decisions are currently largely based, although this number is not informative of treatment responsiveness. However, new approaches are emerging to be able to process these signals to derive meaningful physiological information which could guide therapy selection.

There is a growing body of algorithms emerging to ascertain pathophysiological contributors to sleep-disordered breathing from clinical sleep signals which have been validated against physiological measurements during research sleep studies. Metrics about contributing pathophysiology to OSA have derived from breathing signals for ventilatory control stability [108], the level of respiratory drive which triggers arousal [109], pharyngeal collapsibility and compensatory responses [110, 111]. Individualised information about OSA pathophysiology could be used to select patients for appropriate therapeutic options. For example, a patient with a high contribution of ventilatory instability (high loop gain) in producing OSA, could be steered towards oxygen therapy and away from anatomical treatments which may have limited benefit [65, 112]. The PALM scale (Pcrit, Arousal Threshold, Loop gain and Muscle responsiveness) has been proposed as a potential method to classify OSA patients based on their pathophysiology to suggest suitable treatment modalities [113]. The scale stratifies patients who have a predominant anatomical problem (collapsibility) which likely requires CPAP or mandibular advancement, from those that have significant contributions of non-anatomical pathophysiology and could benefit from a combination of the more experimental therapies targeting these traits. Currently the PALM scale is derived from information acquired from intensive overnight physiological experiments to derive information about contributing pathophysiological mechanisms and therefore cannot be utilised for routine clinical assessment. Algorithms to identify the pathophysiological traits in the PALM scale would bring this a step closer to a clinical tool for matching patients to treatments and if successful, classification of patients using this scale may become a clinical possibility in the future.

A marker of site of pharyngeal collapse has been identified from the nasal flow signal from sleep studies with simultaneous observation using an endoscope [114]. The per cent reduction in inspiratory flow from peak to plateau (amount of negative effort dependence) was used to classify flow shapes. The smallest difference was associated with tongue-related obstruction, moderate with isolated palatal or lateral wall collapse, and severe with epiglottis obstruction. This analysis of airflow signals may therefore give a non-invasive assessment of site of pharyngeal collapse, which may be used to match a patient to a particular therapy most likely to treat that form of obstruction. For example primary oropharyngeal collapse of the pharyngeal airway may be particularly amenable to oral appliance therapy [115]. Knowledge of the primary site of pharyngeal collapse may therefore help selecting patients likely to respond to oral appliances, or for particular upper airway surgeries [66].

Additionally other measures may inform the functional outcomes of therapy. The EEG signals from PSG have also begun to be scrutinised to derive novel and potentially prognostic metrics. For example, a continuous measure of sleep depth (odds product ratio) has been derived [116] with improved sleep quality shown in some people on CPAP therapy, while others actually worsen [117]. This may have implications for therapeutic outcomes and if an individual worsens on a treatment in terms of sleep quality this may require a change in approach. Additionally, the intensity of arousal following respiratory obstruction appears to be a distinct and heritable trait [118, 119]. The heart rate response to arousal is able to be obtained from clinical PSG [120]. This metric, for example, could reflect differences in sympathetic responses and may predict which patients will develop cardiovascular type complications or which patients will respond to OSA therapy for this. Therefore, this analysis of clinical PSG could provide detail on who should be targeted most intensely for OSA therapy.

#### Biomarkers

The definition of a biomarker is a “biological molecule found in blood, other body fluids, or tissues that is a sign of normal or abnormal process, condition or disease” [121]. Since OSA is a complex disorder that has multiple risk factors and consequences, and affects multiple systems, the prospect of a single biomarker for the presence of OSA or signalling susceptibility to specific comorbidities is highly unlikely. Several meta-analyses have recently summarised the current status of the field [122, 123]. Although a large number of studies have assessed biomarkers in OSA, the most promising were considered interleukin-6 (IL-6) and interleukin-10 (IL-10) in adults and a combination of urinary proteins for children [122, 123]. The field may further advance by using ‘omics’ approaches such as metabolomics and proteomics to identify OSA signatures that can be used in diagnosis, identification of susceptibility to comorbidities, and treatment outcomes.

#### Measurement of treatment effectiveness

For a long time treatment success has been defined by the measurement of AHI while the treatment is in use. However, this does not take into consideration the time spent off treatment. This is particularly relevant given the low levels of CPAP usage in the real world, and that often it is removed after only a few hours. This pattern of early removal is particularly concerning given recent associations with OSA in REM and cardiovascular morbidity given that REM sleep is more concentrated towards the later half of the night [124]. Alternate treatments appear to have more favourable compliance profiles (oral appliances) or total compliance (upper airway surgeries), although the AHI reduction may not be complete. Objective evidence for good long-term adherence (> 6 h/night after 1 year of therapy) to OAm has been confirmed through the advent of technology to record hours of usage through temperature-sensing data chips embedded in the appliance [125]. There is increasing awareness that health outcomes are similar despite differences in efficacy between CPAP and OAm, for example [126]. The likely explanation is that these different treatment profiles (moderate efficacy/high compliance and high efficacy/moderate compliance) actually result in the same overall effectiveness [127]. However, this has been a blind spot in the field, particularly as treatment effectiveness is harder to quantify. Metrics have been proposed to take into account total sleep time, time on and off treatment and efficacy, variously termed Treatment AHI, Sleep Adjusted Residual AHI, and Effective AHI [40, 127, 128]. Although these calculations are largely theoretical at this stage as to whether they better reflect health outcomes, these effectiveness metrics may prove to have a role in understanding patient-specific outcomes. A study of partial CPAP users has shown that time off CPAP during the night leaves the patient with significant residual disease (although not necessarily completely back to baseline levels) [128]. Although this has largely been calculated using laboratory PSG, initial comparisons show that home monitoring devices (the WatchPAT in this case) were able to give an equivalent assessment of “effective AHI”. This may prove to be an important consideration in monitoring treatment in individual patients as we move closer to realising the possibility of objective compliance monitoring for non-PAP therapies. In terms of tailoring therapies to OSA patients it is important to think about patient preference and acceptance in the overall assessment of therapeutic effectiveness. This is an area requiring further research to understand the balance between efficacy and effectiveness in individual patients. This highlights the need for patient-centred care approaches and incorporating patient preferences and values and empowering the patient with knowledge to contribute to their own treatment decisions [129].

#### Models of care

As our understanding of the pathophysiology of OSA expands and underpins the development of personalisation strategies, there is a critical need to develop improved models of healthcare delivery that can support the incorporation of this new knowledge into routine clinical care, thereby improving access to care and patient outcomes. Given the high prevalence of OSA there is a need to build capacity in the system, and there is growing evidence supporting the use of simplified models of care involving primary care physicians or nurse practitioners who are supported by access to specialist sleep services when required [130, 131]. The adoption of interdisciplinary models of care within specialist sleep services is also a prerequisite to the translation of personalisation strategies into clinical care. Such models of care should enable selection of tailored diagnostic and therapeutic pathways at the individual patient level.

## Conclusion

The field of sleep medicine is moving into the era of personalised medicine. This means increasing recognition of the limitations of the previous ‘one-size-fits-all’ approaches to treatment and management of OSA. A key part of this is tailoring therapies to the individual. Although CPAP clearly has a place as a highly efficacious treatment, there are new opportunities to tailor therapies to the individual patient. There is a range of current and emerging treatment alternatives to CPAP, however these generally will not be universally efficacious in all OSA patients. This creates the need to match patients to treatments appropriate to their individual pathophysiology in order to maximise treatment response. Tools for elucidating pathophysiological mechanisms and anatomical phenotypes which are amenable to the clinic setting are evolving rapidly and will help provide tailored treatment pathways. Discovery approaches, applied at clinical, electrophysiological, and molecular levels, could bring in a new era of recognisable clinical phenotypes. Future treatment pathways may, therefore, include the ability to determine which OSA patients are susceptible to comorbidity. Furthermore, which individuals will respond to OSA treatment alone vs. those requiring multifaceted treatment strategies, which will require multidisciplinary models of care. Additionally, the framework for understanding treatment response needs to move beyond just a focus on how AHI improves while treatment is in use. Patient engagement and enhancement strategies are needed regardless of which treatment is implemented, and patient participation in these choices are key for optimising real word treatment effectiveness.

## Abbreviations

AHI: Apnoea-Hypopnoea Index
CFD: Computational Fluid Dynamics
CPAP: Continuous Positive Airway Pressure
CT: Computerised tomography
DISE: Drug induced sleep endoscopy
IL-10: Interleukin-10
IL-6: Interleukin-6
ISAC: Icelandic Sleep Apnea Cohort
MRI: Magnetic resonance imaging
mRNA: Messenger RNA
OAm: Mandibular Advancement Appliance
OSA: Obstructive Sleep Apnoea
Pcrit: Critical closing pressure
P-OSA: Positional OSA
PSG: Polysomography
UPPP: Uvulopalatopharyngoplasty
